# Synergistic effects of Tb doping in long-persistent luminescence in Ca_3_Ga_4_O_9_: xBi^3+^, yZn^2+^ phosphors: Implications for novel phosphorescent materials

**DOI:** 10.1016/j.heliyon.2024.e25707

**Published:** 2024-02-02

**Authors:** Stefania Porcu, Franca C. Ugbo, Andrea Pinna, Zaira Carboni, Riccardo Corpino, Daniele Chiriu, Enrico Podda, Pier Carlo Ricci

**Affiliations:** a Department of Physics, University of Cagliari, S.p. no. 8 Km 0700, 09042, Monserrato, CA, Italy; b Centro Servizi di Ateneo per la Ricerca- CeSAR, Università degli Studi di Cagliari, 09042, Cagliari, Italy

## Abstract

Long afterglow phosphors constitute an emerging class of compounds with wide application in several fields, from photonic to dosimetry, solar energy storage and photocatalysis. In this study, we synthesized and thoroughly characterized a new class of persistent emitting materials, Ca_3_Ga_4_O_9_: xBi^3+^, yZn^2+^, zTb^3+^. Through the utilization of X-ray and Raman spectroscopy, as well as optical measurements including static and time-resolved luminescence, thermoluminescence, and phosphorescence, the effects of the Tb concentration on the optical and structural properties of the material has been deeply studied. A suitable mechanism was proposed to account for the long afterglow emission, wherein Tb3+ and Bi3+ ions occupying the Ca2+ sites serve as recombination centers, facilitating the generation of oxygen defects. Zn^2+^ in the Ga^3+^ sites, contribute to the charge balance and generates hole traps in the matrix. The enduring phosphorescence persists for over 3 h following the cessation of UV irradiation, discernible to the naked eye in low-light conditions.

## Highlights

• Efficient phosphorescent material Ca_3_Ga_4_O_9_: xBi^3+^, yZn^2+^, zTb^3+^ has been obtained.
• The emission can be observed for more than 3 h by the naked eye.
• Defect clusters are related to the presence of Tb^3+^.
• Oxygen vacancies are formed close to Tb^3+^ and Bi^3+^ ions.

## Graphical abstract

**Figure:**
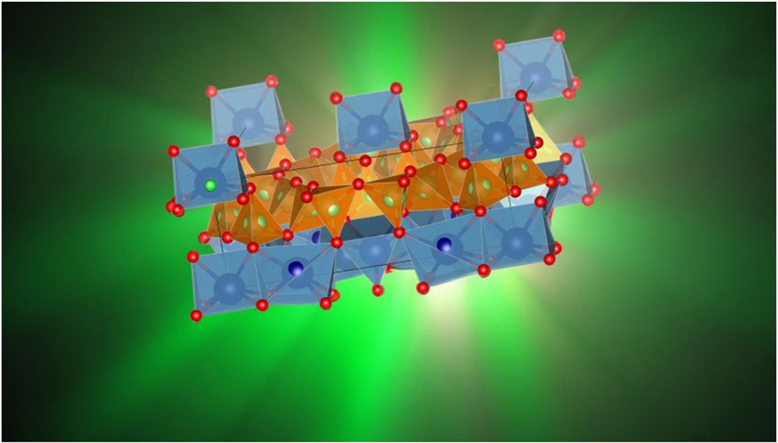

## 1 Introduction

Long Persistent Luminescence Phosphors (LPLPs) are a type of phosphor materials that can store and emit light over a prolonged period after the excitation has been removed, where UV radiation, visible light, X-rays, γ-rays, or electron beam are generally utilized as efficient sources. The emission of light continues for a period ranging from a few seconds to several days and stems primarily from the stored excitation energy within energy traps, which can be liberated through thermal stimulation at room temperature [1]. Due to its remarkable luminescent properties, LPLP has unlocked immense prospects across various application domains. These include security measures and anti-counterfeiting [2], optical information storage [3,4], photocatalysis [5,6], photochemistry [7,8], fingerprint recognition [9], lighting devices [10,11] and potential implementations in synaptic plasticity [12].

Inorganic LPLP are essentially composed of a host matrix, often highly defective, where the localized defects act as trapping centers and are doped with suitable elements to introduce radiative recombination channels. Rare earth elements (Eu^2+,^ Pr^3+^, Ce^3+,^ Eu^3+,^ Tb^3+^) and transition metal (Mn^2+,^ Ti^3+,^ Cr^3+^), are often present in the host matrix as doping luminescence centers providing efficient radiative recombination paths. The duration of the persistent emission is a function of different parameters where the most important are the density of localized trapping sites, the correct ratio between trapping sites and recombination centers and the energy depth of the defects [1]. SrAl_2_O_4_:Eu^2+^, Dy^3+^ [13], ZnGa_2_O_4_:Cr^3+^ [14], Zn_2_GeO_4_:Mn^2+^ [15], are among the most extensively researched and utilized phosphors. However, investigations into the composition, properties, operational principles, and modulation techniques of persistent luminescent materials have led to significant advancements. New long-lasting phosphors have been applied in the near infrared region for night vision or x-ray afterglow imaging [16,17]. Further, the long persistent imaging is strictly connected to the optical stimulated emission technique and related applications [18,19]. In this view, Ca_3_Ga_4_O_9_ represents a promising matrix for optical applications. It has a wide band-gap material (4 eV) with a valence band dominated primarily by the 3p states of Calcium and the 2s 2p states of Oxygen, while the conduction band is dominated by the 4s 4p states of Gallium [20]. The Ca_3_Ga_4_O_9_ crystallites in the Cmm2 space group in the orthographic configuration with cell dimensions of a = 14.374 Å, b = 16.810 Å, and c = 5.3082 Å.

Bi^3+^ doped Ca_3_Ga_4_O_9_ shows a broad emission in the visible region with excitation channels at 280 nm and at 350 nm [21]. The deep UV channel is related to a band-to-band excitation, while the band in the UVC region represents a direct excitation of the Bismuth ions [21]. Due to charge compensation effect, co-doping with Zn ions enhances the optical performance of Ca_3_Ga_4_O_9_: Bi^3+^, by increasing the cyan emission [22].

Ca_3_Ga_4_O_9_ represents a suitable matrix for rare earth substitution in the Ca sites and samples doped with Eu or Tb have shown properties as red and green phosphors for white LED applications [20,23]. Its application as LPLP has been suggested but its potential was not already fully exploited.

A recent paper [20] discusses the optical characterization of Ca_3_Ga_4_O_9_ doped with Tb and Zn. The presence of Zn^2+^ enhances photoluminescence (PL) and persistent luminescence (LPL) properties by reducing lattice symmetry and increasing the number of intrinsic sites. Furthermore, co-doping with Tb^3+^ and Zn^2+^ leads to a further enhancement in the densities of traps located at different depths.

In this study, we have effectively investigated the application of a Bi, Zn, and Tb-doped Ca_3_Ga_4_O_9_ (hereafter CGO) matrix as a highly efficient long persistent phosphor. Through structural characterization techniques such as X-ray and Raman spectroscopy, as well as analysis of optical properties including static and time-resolved luminescence, thermoluminescence, and phosphorescence, we have gained a comprehensive understanding of the material's optical properties. Furthermore, these findings contribute to the development of a valuable model for elucidating the recombination paths within the material, underling the fundamental role of the charge transfer processes between the intragap levels of the defect site and recombination centers.

## 2 Materials and methods

### 2.1 Materials

CaCO_3_ (CAS No.: 471-34-1), Ga_2_O_3_ (CAS No.: 12024-21-4), Bi_2_O_3_ (CAS No.: 1304-76-3), ZnO (CAS No.: 1314-13-2), and Tb_4_O_7_ (CAS No.: 12037-01-3) were purchased from Sigma Aldrich. All chemicals were used as received without further purification.

### 2.2 Synthesis procedure

The Ca_3_Ga_4_O_9_: xBi^3+^, yZn^2+^, zTb^3+^ samples were prepared by a high-temperature solid-state synthesis.

3CaCO_3_ + 2Ga_2_O_3_ + xBi_2_O_3_ + yZnO + zTb_4_O_7_ → Ca_(3−x−z)_Ga_(4−y)_O_9_:Tb^3+^

Due to the similarity of their ionic radii, the Bi^3+^ cation (r = 1.03 Å) substitutes for the Ca^2+^ cation (r = 1.00 Å) in the crystal structure. Similarly, the Tb^3+^ cation (r = 0.92 Å) substitutes for the Ca^2+^ cation. As for the Zn^2+^ cation (r = 0.6 Å), having an ionic radius more similar to that of the Ga^3+^ cation (r = 0.47 Å), it was substituted for the latter.

The precursors, in the correct amounts, were first mixed in an agate mortar and then subjected to a heat treatment of 1200 °C for 10 h. For the synthesis of the materials, the percentages of Bi^3+^ and Zn^2+^ were kept constant (2 % and 3 % respectively) while the doping percentages with Tb^3+^ were varied between 2 % and 8 % wt.

### 2.3 Characterization techniques

Powder X-ray diffraction data were obtained using a PANalytical X'Pert Pro diffractometer equipped with an X'Celerator detector. The measurements were conducted in the reflection geometry Bragg−Brentano θ–θ geometry, employing Cu-Kα1 radiation (λ = 1.54060 Å) within the 10−80° (2θ) range, with a step size of 0.05° (2θ) and operating at 40 kV/40 mA.

Phase identification was carried out by means of PDF-2 database and the selected crystal structures were retrieved from the Cambridge Crystallography Data Centre (CCDC) from: https://www.ccdc.cam.ac.uk/structures/

Raman spectroscopy measurements were conducted using an MS750 spectrograph (Sol-Instruments) fitted with a 600 gr/mm grating. A 785 nm laser served as the excitation source, focused via a 10× Olympus objective lens, with an approximate laser power of 1x10-2 W/cm2. No discernible heating effect on the samples was observed. The measurements were carried out at room temperature, with a spectral resolution of 1 cm^−1^.

Steady time photoluminescence measurements were conducted using an MS750 spectrograph (Sol-Instruments) fitted with a 175 gr/mm grating. A 405 nm laser served as the excitation source, directed through a 10× Olympus objective lens.

For time-resolved luminescence measurements, an optical parametric oscillator with a frequency doubler device was utilized, driven by the third harmonic (355 nm) of a pulsed Nd: YAG laser (Quanta Ray Pro 730). The excitation pulse had a width at half-maximum of 8 ns, a repetition rate of 10 Hz, and a spectral bandwidth of less than 0.3 cm−1. Signals were captured using a spectrograph (Arc-SpectraPro 300i) with a spectral bandpass of less than 2.5 nm and detected by a gateable intensified CCD (PI MAX Princeton Inst.). To minimize dark current, the detector was cooled to −20 °C using a Peltier device.

*Thermoluminescence measurements* were performed using a thermoluminescence system composed by an heating controller with programmable power supply (Agilent 6030A), 0–200V/0-17A, 1000W and Phototube detector coupled with a high sensitivity data acquisition and control unit HP 3852A. The measurements were performed in a range between RT and 523 K with a heating rate of 10 °C/min controlled by LabVIEW 2019.

*Phosphorescence measurements* were performed at 20 °C monitoring the overall emission with accumulation time of 1 s by using an Avantes Sensiline Avaspec-ULS-TEC Spectrometer.

## 3 Results and discussion

**Fig. 1:**
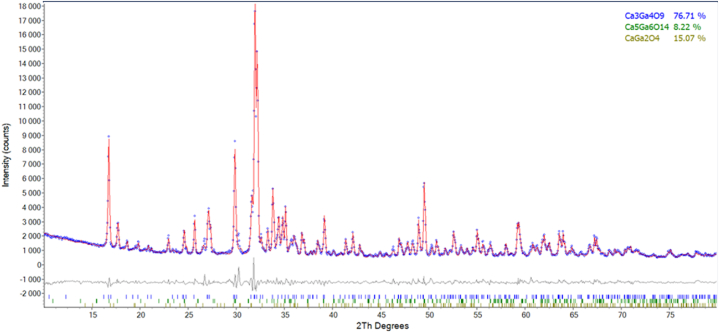
Powder X-Ray Diffraction analysis on sample **1** showing: experimental data (blue dots), calculated pattern (red), and the difference curve (grey). Vertical tick marks at the bottom indicate positions of the allowed hkl reflections for each crystal phase labelled in the upper right corner. (For interpretation of the references to colour in this figure legend, the reader is referred to the Web version of this article.)

Ca_3_Ga_4_O_9_, belongs to the Cmm2 space group in the orthorhombic configuration where the GaO_4_ tetrahedra and calcium cations are arranged in layers. There are four crystallographic sites for Ca^2+^, Ca1, Ca2, Ca3, and Ca4. The Ca1 and Ca3 atoms are arranged in an orthorhombic configuration surrounded by six oxygen atoms, while Ca2 and Ca4 are surrounded by eight oxygen atoms [24]. The refined diffraction pattern of the synthetized samples (Fig. 1 for CGO: 3 % Bi^3+^, 2 % Zn^2+^ and Fig. S1 for the Tb doped samples) show the presence of relative residuals mainly attributed to the observed minority phase peaks but exhibits excellent agreement between the observed and calculated diffraction pattern. Phase identification was carried out by means of PDF-2 database and the selected crystal structures were retrieved from the Cambridge Crystallography Data Centre (CCDC). Quantitative phase analysis for the experimental XRD pattern was performed via a Rietveld analysis using TOPAS v6 which allowed for the quantification of the predominant crystalline phases: Ca_3_Ga_4_O_9_ (76.7 %), CaGa_2_O_4_ (15.1 %), Ca_5_Ga_6_O_14_ (8.2 %). The peak shapes were described by the Fundamental Parameters (FP) approach implemented in TOPAS and a satisfactory model was obtained with a final Rwp = 7.12 %. No significant variation has been observed in the main phases with respect Matrix (CGO: 2 % Bi^3+^, 3 % Zn^2+^) and no agglomerated or residues from the Tb precursor have been observed.

Ca_3_Ga_4_O_9_, and the secondary phases found in the compounds triply doped, CaGa_2_O_4_, and Ca_5_Ga_6_O_14_ are all orthorhombic and belong to the cmm2, pna21, and Cmc21 space groups, respectively [25,26]. While Ga occupies the tetrahedral site in all three structures, the main distinctions lie in the surrounding oxygen arrangement with respect to Ca. In Ca_3_Ga_4_O_9_, there are two equivalent sites with six and eight oxygens; in CaGa_2_O_4_, Ca occupies two sites, both five-coordinated (with different Ca–O distances); whereas in Ca_5_Ga_6_O_14_, Ca occupies only one six-coordinated site.

The synthesis conditions for all three structures are very similar. They were obtained using the same precursors (CaCO_3_ and Ga_2_O_4_) through a solid-state reaction at comparable temperatures (1200 °C for 10 h–1300 °C for 6 h and 1200 °C for 6 h, respectively) [[25], [26], [27]]. The variation lies in the stoichiometry between the starting precursors. Our synthesis with one or two doping elements demonstrates a high percentage of achieving the pure phase (Ca_3_Ga_4_O_9_), as reported in several previous studies [20,21,[25], [26], [27]]. However, in the triple-doped matrix, we have never achieved a percentage higher than 75–76 % of the pure phase. The most likely reason is the elevated percentage of defects and the subsequent formation of local stoichiometric variations from the initial composition.

Further, the Bi emission strongly depends on the local crystal field. In Ca_3_Ga_4_O_9_, the Bi emission is centered at 538 nm [21], in CaGa_2_O_4_ the emission is red-shifted to 580 nm [25], while in Ca_5_Ga_6_O_14_, it is blue-shifted to 498 nm [27]. The emission observed in the triple-dopant compounds overlaps the spectrum of the Bi in the pure matrix and no components at lower or higher energies have been observed in the luminescent measurements.

**Fig. 2:**
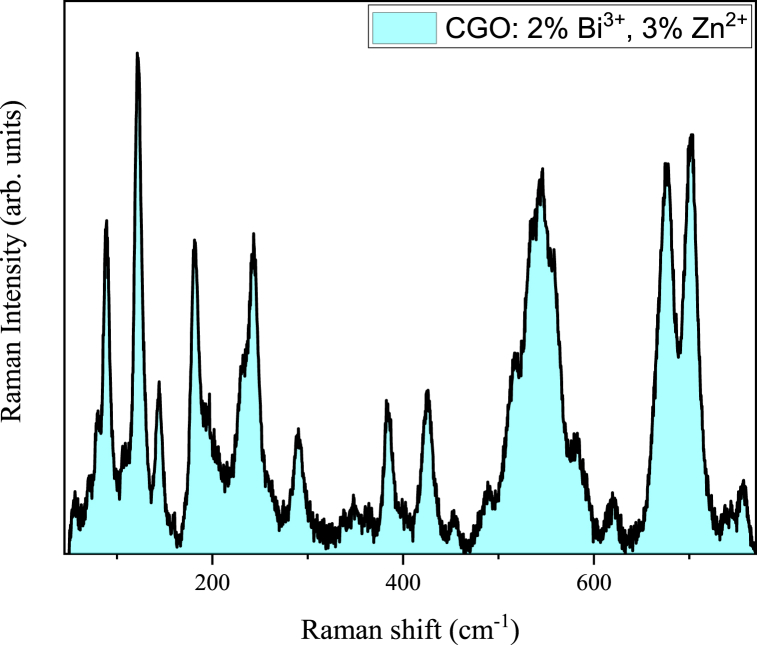
Raman spectrum of CGO: 2 % Bi^3+^, 3 % Zn^2+^ matrix.

The Raman spectrum of the CGO: 2 % Bi^3+^, 3 % Zn^2+^ matrix (hereafter CGO: 2 % Bi^3+^, 3 % Zn^2+^) is reported in Fig. 2, providing further insights into both the structural and vibrational properties. It comprises of numerous sharp bands covering the spectral range between 50 cm^−1^ to 800 cm^−1^. The most prominent bands reside at 121 and 545 cm^−1^, with a broad structure with two main peaks centered at 675 cm^−1^ and 702 cm^−1^, respectively. Intense bands are located at 89, 180, 243 cm^−1^, in conjunction with several other weaker but sharp bands. The band at 545 cm^−1^ have shoulders at both the side (approximatively at 485, 515 and 580 cm^−1^).

Since no Raman data or computational analysis on this compound has been published to date, the assignment of the main vibrational modes has been performed by the studies of similar structures and on the general position on the bands, however a detailed analysis is mandatory, but it remains out of the scope of the present work.

The Ca_5_Ga_6_O_14_ compound features an orthorhombic structure, akin to Ca3Ga4O9, characterized by layers of tetrahedral rings as its primary structural motif. Both compounds can be conceptualized as structures composed of tetrahedral layers, with calcium cations positioned between these layers. Within these layers, Ga^3+^ ions form four-coordination (GaO_4_) arrangements, while Ca^2+^ ions exhibit six-coordination (CaO_6_) with the surrounding O^2−^ ions [28]. Further, in the Ca_3_Ga_4_O_9_ structure, there are two additional Ca sites bonded to eight oxygen (CaO8) in a larger environment. The GaO_4_ tetrahedra generate the band at 545, 675 and 757 cm^−1^ (bending and stretching of GaO_4_ units) [29]. Further assignments concern the modes at lower wavenumber that involve the collective motion O-Ga-O among different tetrahedra structures and the mode at 121 cm^−1^ ascribed to the Ca–O bond [30]. Finally, the intense modes at 290 and 702 cm^−1^ are assigned to the bending and stretching vibrational mode of the Ca–O8 units [28].

**Fig. 3:**
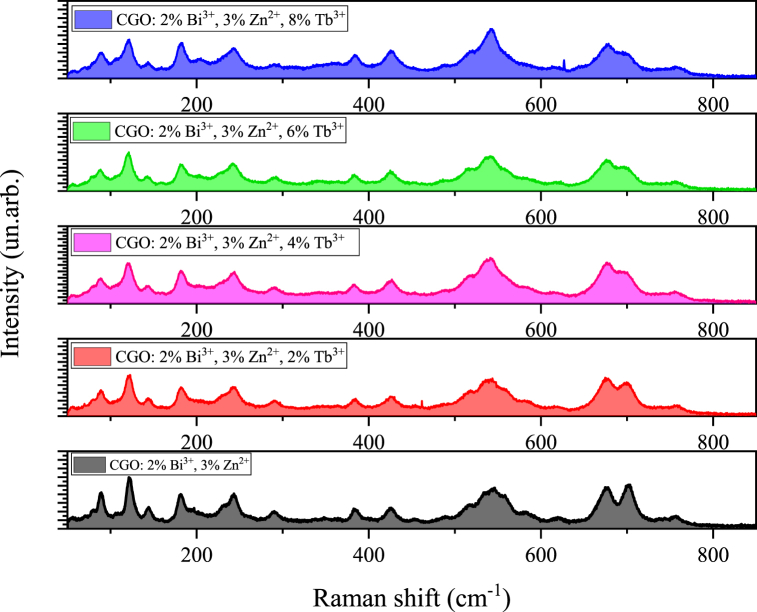
Raman spectra of CGO: 2 % Bi^3+^, 3 % Zn^2+^ doped with different percentages of Terbium.

This last mode suffers of the main variation with the increasing with Tb doping. In Fig. 3 it can be observed that the increase of Tb ions produces a significant decrease of the relative Raman intensity with respect the vibration of the GaO_4_ units at 545 cm^−1^ (from 1.08 in the CGO matrix down to 0.54 in the sample Tb doped with the highest percentage) and a shift to the lower wavenumbers (703 cm^−1^ to 698 cm^−1^). Both the effects could be due to the distortion Ca–O8 units due to the formation of oxygen vacancies generated to balance the different oxidation state between Ca^2+^ and Tb^3+^ and for the different ionic mass (40–170.9) [31,32].

The Raman spectrum of the Bi and Zn co-doped sample (hence without the further doping with Tb ions) does show similar variation with respect the undoped sample.

**Fig. 4:**
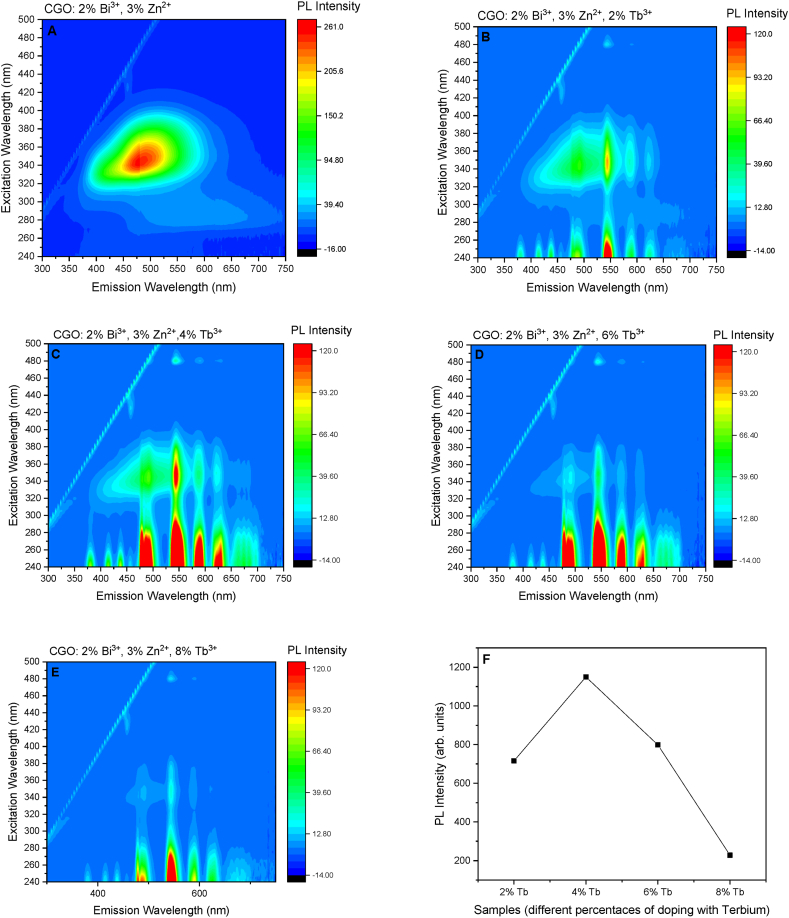
3D plot of the excitation and emission properties of the different samples: A) CGO: 2 % Bi^3+^, 3 % Zn^2+^; B) CGO: 2 % Bi^3+^, 3 % Zn^2+^, 2 % Tb^3+^; C) CGO: 2 % Bi^3+^, 3 % Zn^2+^, 4 % Tb^3+^; D) CGO: 2 % Bi^3+^, 3 % Zn^2+^, 6 % Tb^3+^; E) CGO: 2 % Bi^3+^, 3 % Zn^2+^, 8 % Tb^3+^. Panel F reports the intensity of the 545 nm emission upon 250 excitation wavelength.

The effects of the different dopants are clearly visible in the 3D plot of the excitation and emission properties of the different samples (Fig. 4A,B,C,D,E). The Bi^3+^ ions generate an intense and broad luminescence band at a about 530 nm, with the main excitation in the UV range (350 nm), clearly visible in the Bi:Zn codoped sample [21] (Fig. S2). The Tb doped samples show the characteristic Tb emissions band at 478 nm, 542 nm, 586 nm e 621 nm, due to the recombination from the ^5^D_4_ to the ^7^F_j_ levels (J = 3 to 6, respectively) with excitation in the deep UV range and a minor excitation band at 350 nm in the samples with Bi and Zn as co-dopant with broad emission overlapped to the narrow emission from the Tb ^5^D_4_ levels (Fig. S3). The excitation spectra of the sample single Tb doped present only the band at 250 nm and no broad emission are detected. The Tb sample with only Bi or Zn as co-dopant still present mainly the excitation of single Tb doped sample with minor direct effects in the optical properties (Fig. S4). It is worth noting that the overall intensity of the luminescence at the Tb^3+^ sites does not grow asymptotically but it presents a maximum for the sample with 4 % Tb doping concentration (Zn 3 %, Bi 2 %) (Fig. 4F).

**Fig. 5:**
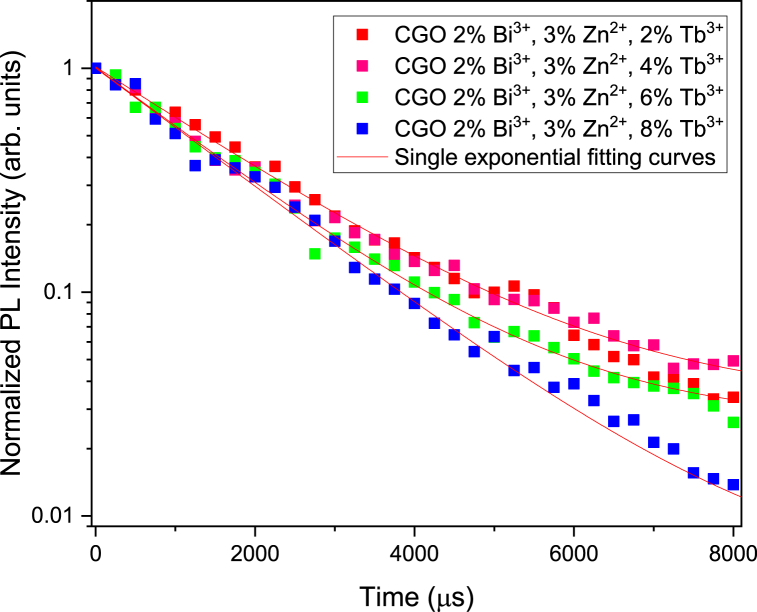
Time decay monitored at 545 nm showing the dependency on Tb concentration.

The time evolution of Tb luminescence, observed at 545 nm and accurately depicted by a single exponential curve, reveals a subtle dependency on Tb concentration (Fig. 5). The observed time decay constants range between 1850 μs (2 %) and 1600 μs (8 %), suggesting the presence of negligible concentration quenching effect connected to energy transfer mechanism among the rare earth element ions [33].

**Fig. 6:**
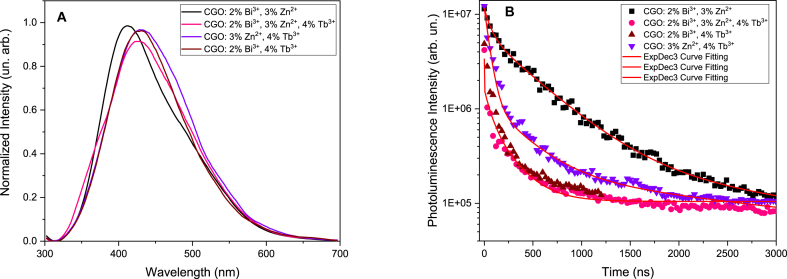
(A) Spectrally resolved emission with excitation at 250 nm, time delay 20 ns, time window 20ns. (B) Time decay monitored at 450 nm.

To comprehensively characterize the radiative kinetics of the samples, time-resolved measurements were conducted within a shorter time domain, specifically in the nanosecond regime (see Fig. 6). Under these conditions (time delay: 20 ns, time window: 20 ns), a broad emission around 450 nm is evident in all samples, regardless of doping ions or co-doping conditions (CGO: 2 % Bi^3+^, 3 % Zn^2+^, 4 % Tb^3+^; CGO: 2 % Bi^3+^, 3 % Zn^2+^; CGO: 3 % Zn^2+^, 4 % Tb^3+^; CGO: 2 % Bi^3+^, 4 % Tb^3+^). This emission is directly attributed to the presence of defect sites, as illustrated in Fig. 6A.

The temporal behavior of this broad emission, monitored at the central wavelength of 450 nm, exhibits a faster response in samples containing Tb (see Fig. 6B), suggesting a competitive non-radiative charge transfer process to the Tb sites. It is noteworthy that in the nanosecond range, emissions from Tb ions do not appear (at a delay time of 20 ns), and the well-defined broad band centered around 450 nm dominates.

To better underline the different temporal behavior, we fit the experimental data with triple-exponential function and we indicate the average lifetime as [34]:

$$\bar{\tau }=\frac{A_{1}\tau_{1}^{2}+A_{2}\tau_{2}^{2}+A_{3}\tau_{3}^{2}}{A_{1}\tau_{1}+A_{2}\tau_{2}+A_{3}\tau_{3}}$$

Where τ_i_ stands for the fitting lifetime and A_i_ the relative constant for the curve:

$$I_{t}=I_{0}+\sum_{1}^{3}A_{i}e^{-\frac{t}{\tau_{i}}}$$

And I_t_ represents the time dependent luminescence intensity and I_0_ the initial luminescence intensity. The average time decay constants are 1.12 μs, 0.87 μs 195 and 189 μs for the samples CGO: 2 %Bi^3+^,3 %Zn^2+^, CGO: 3 %Zn^2+^, 4 %Tb^3+^; CGO: 2 %Bi^3+^, 4 %Tb^3+^, and CGO: 2 %Bi^3+^, 3 %Zn^2+^, 4 %Tb^3+^, respectively.

As previously highlighted, the recombination from the D level of Tb exhibits a time decay constant on the order of hundreds of microseconds to milliseconds and remains undetected in the short time-scale domain.

**Fig. 7:**
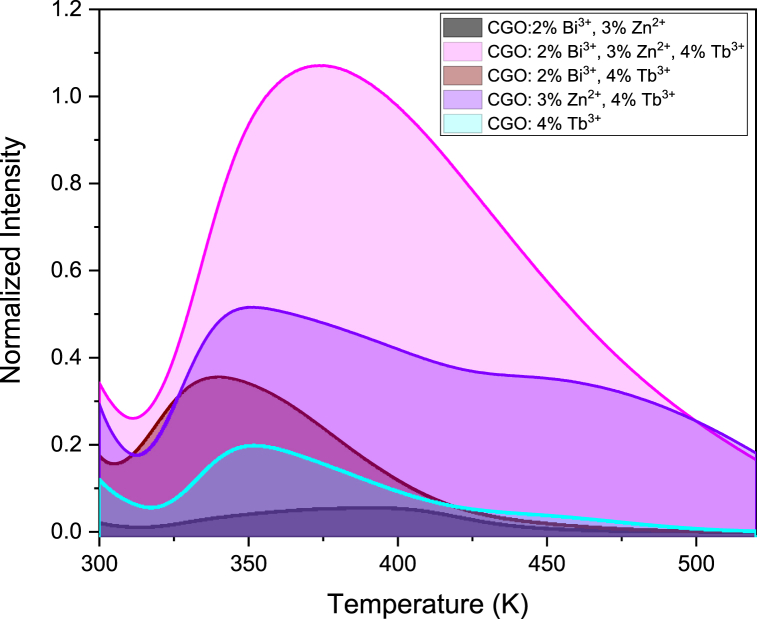
Thermoluminescence measurements of CGO doped and co-doped samples.

Thermoluminescence measurements give important insights on the effect of the dopant ions in the matrix and about defects energy dept in the host band gap. Fig. 7 reports the glow curves of CGO samples, doped and co-doped with Bi, Zn and/or Tb after excitation at 250 nm. The measurements have been performed with the same experimental parameters and the same amount of powder has been utilized for each sample to compare the overall efficiency of the Thermoluminescence curves. The sample CGO: 2 % Bi^3+^, 3 % Zn^2+^ free of Tb ions, presents the lowest thermoluminescence efficiency while the triple doped sample possesses the highest intensity. However different shape and peak positions have been observed and a detailed analysis is required.

**Fig. 8:**
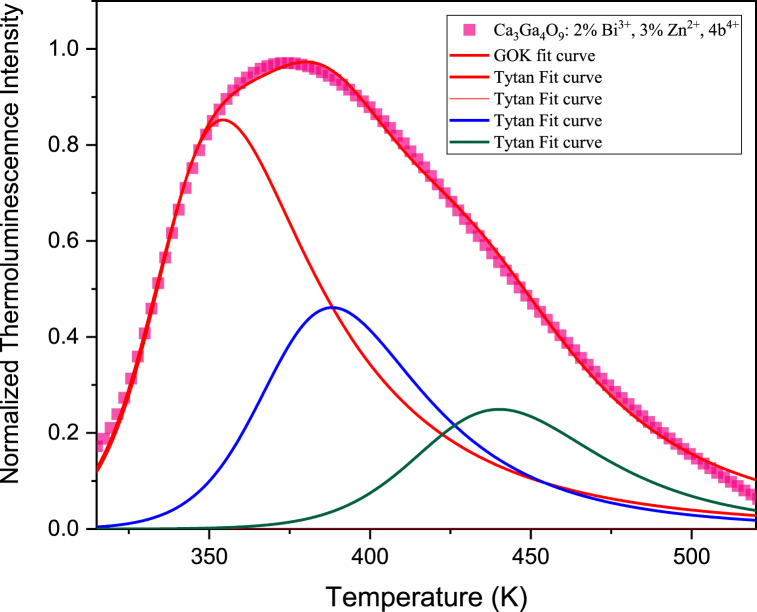
Thermoluminescence curve of CGO: 2 % Bi^3+^, 3 % Zn^2+^, 4 % Tb^3+^and curve fit by using the generalized order of kinetic model.

The shape and the peak position of the thermoluminescence curves depends on several parameters: the depth of the defect center, the possibility of re-trapping after the thermal release at the same site, the involvement in the process of electron and/or hole and, further, some experimental parameter like the heating rate. Henceforth, for deeper insights, we have employed the Generalized Order of Kinetic Model (GOK) to analyze the thermoluminescence (TL) experimental curve. In this model, we postulate that the thermal response can be delineated by the superposition of three components, as depicted by the following equation: (Fig. 8) [35,36]:

I(T)=∑J=1nAj∙SjeEjkT∙[I∙(bj−1)Sjβ∫t1t2eEjkTdt]bjbj−1

A, a constant of proportionality, varies according to the concentration of traps. E represents the depth of thermal traps, denoting the energy required to release trapped charge carriers, which may differ from the optical energy necessary for their release. K stands for Boltzmann's constant, while b and S denote parameters associated with trapping kinetics and the frequency factor, respectively. β indicates the sample heating rate, set at 10 K/s. Higher-order kinetics parameters typically emerge when there is a heightened likelihood of re-trapping released electrons compared to their recombination at the luminescent center. Meanwhile, the frequency factor S, or the 'attempt-to-escape' frequency, quantifies the frequency of bound electrons interacting with lattice phonons per second.

The Table 1 reports the values for obtained by the fitting procedure applied to the thermoluminescence curves reported for CGO samples doped and co-doped with Tb, Bi and/or Zn, pointing out the energy of the traps at 0.85, 0.88 and 0.91 eV, denominated 1, 2, 3, respectively (Fig. 8 and Fig. S5). The bands at 1 and 2 are common in all the samples with Tb or Bi while the band C is related to the presence of Zn (see the fitting curves in Fig. S5). On this view, we assigned to hole traps at the Ga vacancies the 3 band [20], and electron trapping sites related to the substitution of Ca with trivalent ions Tb ions and Bi respectively for the trapping site A and B.

**Table 1:** Fit parameters of CGO samples thermoluminescence spectrum.

Sample	A_1_	b_1_	S_1_ (s^−1^)	E_1_ (eV)	A_2_	B_2_	S_2_ (s^−1^)	E_2_ (eV)	A_3_	B_3_	S_3_ (s^−1^)	E_3_ (eV)
CGO: 4 %Tb^3+^	75	3.7	3E10	0.85	24	4	7E9	0.88	–	–	–	–
CGO:2 % Bi^3+^, 4 %, Tb^3+^	130	3.8	9E10	0.85	35	3.2	2E10	0.88	–	–	–	–
CGO: 3 % Zn^2+^, 4 % Tb^3+^	180	3.8	6E10	0.85	170	4.2	3E9	0.88	205	4.8	8E7	0.92
CGO: 2 % Bi^3+^, 3 % Zn^2+^, 4 % Tb^3+^	409	4.1	2E10	0.85	205	3.2	3.8E9	0.88	122	2.7	3E8	0.91

**Fig. 9:**
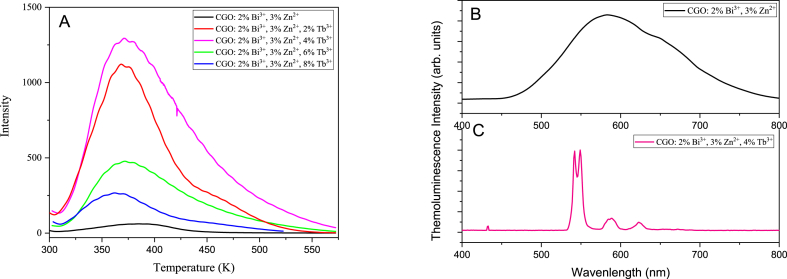
(A) Thermoluminescence curves of CGO samples with different Tb content; Spectrally resolved thermoluminescence curve at 370 K of the sample (B) CGO: 2 % Bi^3+^, 3 % Zn^2+^ and (C) CGO: 2 % Bi^3+^, 3 % Zn^2+^, 4 % Tb^3+^.

The function of Tb within the CGO matrix remains consistent with that of Bi due to their similar ionic radii, equivalent valence charges, and consequentially, their occupation of the same substituting site. The thermoluminescence curves of the co-doped CGO: 2 % Bi^3+^, 4 % Tb^3+^ and CGO: 4 % Tb^3+^, show a similar behavior but with a significant increase of the intensity in the single Tb doped sample, while the role of Zn appears different, since it acts the Ga site. When the trivalent ions Tb ion and/or Bi ion occupies the position of the Ca^2+^ site, the charge is not balanced inducing the distortions in the local symmetry, that could be balanced by presence of Zn^2+^ in Ga^3+^ site, but inducing new hole trapping site in matrix [22]. The fully doped sample (Zn, Bi and Tb) provides the thermoluminescence curve with the highest intensity. Two factors actively contribute to the increase of efficiency: the higher concentration of dopants (and defect sites) and the increased efficiency in the radiative recombination path in the rare earth (Fig. 9). Increasing the Tb content in the host matrix, the glow peak in the thermoluminescence measurements produce a variation in the overall intensity without altering the curve shape and hence the defect types and relative ratio among them.

Similarly, to the PL measurements, the 4 % Tb^3+^ doped sample has highest emission intensity, since the resonant charge transfer among the Tb ions produces the progressive intensity decrease. It is worth to pointing out that the optical emission take place at the Tb site as evidenced by the spectrally resolved thermoluminescence curve of the sample co-doped with Tb respect the sample without the rare earth (Fig. 9B and Fig. C). Bi^3+^ and Tb^3+^ substitution contribute to the formation of defect clusters in the proximity of the dopant: Tb^3+^_Ca_–V_0_ and Bi^3+^_Ca_–V_0_ acting as electron trapping centers with both Tb^3+^ and Bi^3+^ as the emission centers. The different charge balance generates Oxygen vacancies in the host matrix and the formation of F+ center or F center, depending on the number of electrons trapped in the vacancy sites [37]. Due to the deeper trapping potential experienced by electrons in F+ centers compared to those in F centers within oxides, the thermoluminescence curve typically exhibits two peaks [37]. Additionally, the formation of clustered defects promotes the occurrence of the tunnelling process, which is contingent upon the distance between the involved centers and, consequently, the doping percentage. Conversely, as the ionizing potential of a dopant cation decreases, its ability to delocalize electron density to stabilize an empty anion vacancy increases. Therefore, it is anticipated that Tb exhibits a stronger propensity to attract an anion vacancy (oxygen defect cluster) compared to Bi3+ [38,39]. Consequently, it is expected the formation of defect clusters in the proximity of the Tb sites. The stronger effect of the Tb doping is evidenced in the analysis of the Raman spectra, too. As already discussed, the decrease in intensity of the vibrations related to the Ca–O_8_ units is much more pronounced in the Tb doped samples, while few or neglecting effects have been observed in absence of the rare earth.

**Fig. 10:**
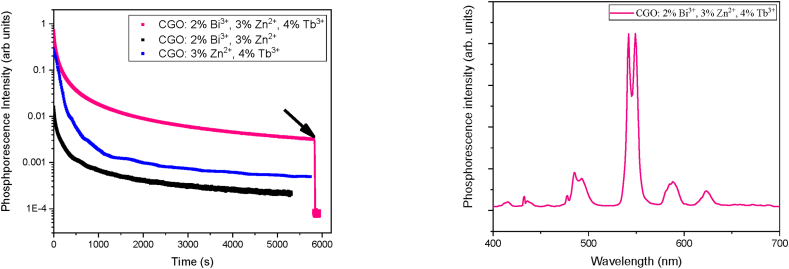
(A) Long-lasting phosphorescence of CGO: 2 % Bi^3+^, 3 % Zn^2+^, 4 % Tb^3+^, CGO: 2 % Bi^3+^, 3 % Zn^2+^, and CGO: 3 % Zn^2+^, 4 % Tb^3+^. The arrow indicates when the phosphorescence is interrupted. (B) Spectrally resolved emission of the luminescent persistence for CGO: 2 % Bi^3+^, 3 % Zn^2+^, 4 % Tb^3+^.

Fig. 10A illustrates the long-lasting phosphorescence curves exciting at 250 nm for three samples: the triple-doped CGO: 2 %Bi^3+^, 3 %Zn^2+^, 4 %Tb^3+^; the double-doped CGO: 3 %Zn^2+^, 4 %Tb^3+^; and the CGO: 2 %Bi^3+^, 3 %Zn^2+^. It is important to underline that no phosphorescence has been observed by excitation in the PLE excitation peak at 350 nm). The fast initial decline followed by slow decays, indicates the existence of both shallow and deep traps [16], as already indicated by the analysis of the thermoluminescence measurement.

The overall emission has been collected, even though the afterglow emission peaks differ (see Fig. 9B and figure C). A comparison of the curves underscores the increase in defect sites due to the presence of Bi and the synergistic effect with Tb ions (see for comparison the long-lasting emission from the double-doped CGO: 3 %Zn^2+^, 4 %Tb^3+^). It further highlights the higher efficiency of the Tb-doped samples compared to those doped solely with Bi and/or Zn. After approximately 3 h from illumination, the phosphorescence intensity of the triple-doped sample exceeds two orders of magnitude compared to dark conditions. Additionally, it is essential to note that the spectral shape remains unchanged, resembling the recombination at the Tb site (see Fig. 10B). No signal in the thermoluminescence curve after the LPL decay has been detected showing that the defect sites are in contact each other.

**Fig. 11:**
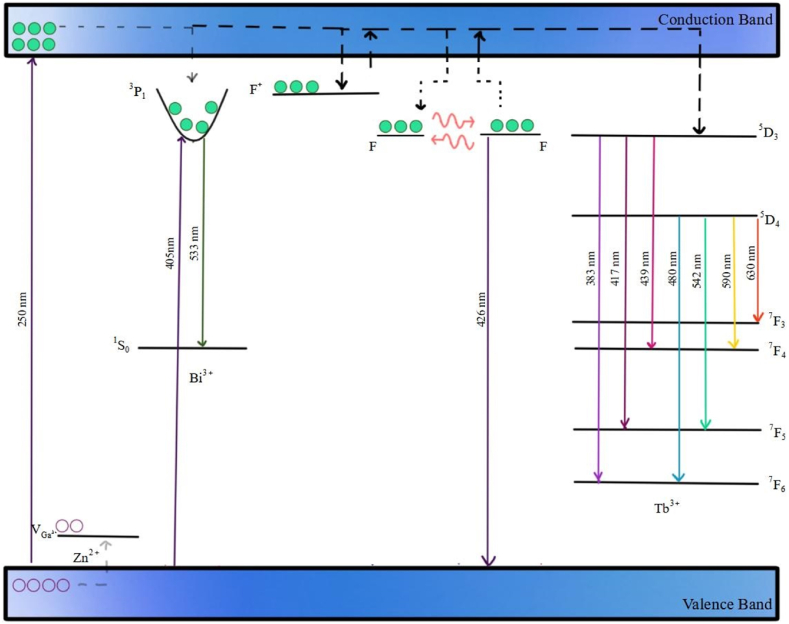
Scheme of the optical process in the CGO doped with Zn, Bi and Tb.

Analysis of experimental results allows for the formulation of an optical process scheme within the CGO matrix triply doped with Zn, Bi, and Tb, as illustrated in Fig. 11.

Under band-to-band excitation (250 nm), electrons from the valence band and the ground level of Ga^3+^ are photoexcited to the conduction band and subsequently stored in the F and F+ centers. Concurrently, holes are stored in Gallium vacancy sites, linked to the presence of Zn as a dopant. These defect centers are connected to the conduction band at room temperature, contributing to long phosphorescence. Upon exposure to thermal energy, the traps are further emptied, as depicted in the thermoluminescence curve. The F centers generate the shallow thermoluminescence bands 1 and 2 (refer to Table 1), while the hole traps are associated with the deeper band 3. All contribute to the long-lasting emission observed at elevated temperatures.

Due to the lower ionizing potential of Tb, defect centers are primarily localized near the Tb site, facilitating efficient recombination from the D_3_ and D_4_ levels, as evident in the typical green emission spectra of Tb in phosphorescence, thermoluminescence, and time-resolved measurements in the microsecond domain. Recombination at the Tb^3+^ sites compete with the Bi sites but, owing to a higher probability of forming a Tb^3+^_Ca_–V_0_ cluster (refer to Raman spectra), it exhibits greater efficiency. Bi^3+^ as a co-dopant element increases the overall defectiveness of the samples and, consequently, the storing capacity of photogenerated carriers, as seen in thermoluminescence curves and the long-lasting emission of the triple-doped sample compared to the sample without Tb. Time-resolved measurements in the nanosecond domain indicate that F centers are non-radiatively connected with Tb centers, while no direct energy transfer to Bi ions can be confirmed.

The provided scheme aims to offer a general overview of the storage mechanism in a new material with increased phosphorescence capability, attributed to the presence of Tb ions in the matrix.

### 3.1 Conclusion

In conclusion, by synthesizing and thoroughly characterizing Ca_3_Ga_4_O_9_: xBi^3+^, yZn^2+^, zTb^3+^ phosphors doped with different amounts of Tb^3+^ ions, we were able to gain a deep understanding of the effects of these dopants on the material's optical and structural properties.

Our findings suggest that defect clusters, specifically oxygen vacancies and Tb^3+^ ions, play a crucial role in the phosphor's behavior. Oxygen vacancies act as electron trapping centers, while Tb^3+^ ions act as hole trapping centers. Furthermore, we propose a mechanism for the tunable long afterglow emission, where Tb^3+^ and Bi^3+^ in the Ca^2+^ sites act as recombination centers, promoting the formation of oxygen defects. Additionally, Zn^2+^ in the Ga^3+^ sites contribute to charge balance and generates hole traps within the matrix. The result of this intricate interplay is a remarkable long-lasting phosphorescence, observable by the naked eye for up to 3 h in a dark environment after discontinuing UV irradiation. These findings provide valuable insights into the underlying processes and mechanisms involved in achieving prolonged afterglow in phosphor materials. Overall, the comprehensive understanding gained from this study enhances the potential applications of long afterglow phosphors and sets the stage for further advancements in this field.

## Data availability

Data will be made available on request.

## CRediT authorship contribution statement

**Stefania Porcu:** Writing – original draft, Methodology, Investigation, Formal analysis. **Franca C. Ugbo:** Investigation, Formal analysis. **Andrea Pinna:** Methodology, Investigation, Formal analysis. **Zaira Carboni:** Investigation, Formal analysis. **Riccardo Corpino:** Investigation, Formal analysis. **Daniele Chiriu:** Investigation, Formal analysis. **Enrico Podda:** Investigation, Formal analysis. **Pier Carlo Ricci:** Writing – review & editing, Writing – original draft, Methodology, Investigation, Conceptualization.

## Declaration of competing interest

The authors declare that they have no known competing financial interests or personal relationships that could have appeared to influence the work reported in this paper.
