# Supplementary material for: Synergistic effects of Tb doping in long-persistent luminescence in Ca3Ga4O9: xBi3+, yZn2+ phosphors: Implications for novel phosphorescent materials
Source: Heliyon. 2024 Feb 2;10(3):e25707. doi: 10.1016/j.heliyon.2024.e25707 (PMC10850978; doi:10.1016/j.heliyon.2024.e25707)
Supplement: Multimedia component 1 [file mmc1.docx]

**Supporting Information**

**Synergistic Effects of Tb doping in Long-Persistent Luminescence in Ca_3_Ga_4_O_9_: xBi^3+^, yZn^2+^ Phosphors: Implications for novel phosphorescent materials**

Stefania Porcu^a^, Franca C. Ugbo^a^, Andrea Pinna^a^, Zaira Carboni^a^, Riccardo Corpino^a^, Daniele Chiriu^a^, Enrico Podda^b^, Pier Carlo Ricci^a*^

^a^ Department of Physics, University of Cagliari, S.p. no. 8 Km 0700, 09042 Monserrato, CA, Italy

^b^ Centro Servizi di Ateneo per la Ricerca- CeSAR, Università degli Studi di Cagliari, 09042 Cagliari, Italy

***Corresponding author carlo.ricci@dsf.unica.it**

S1. XRD diffraction patterns of Tb doped samples

Figure S2 - Emission spectrum of CGO: 2%Bi ^3+^, 3% Zn^2+^. Excitation 350 nm

Figure S3 Emission spectra of CGO: 2% Bi^3+^, 3% Zn^2+^ doped with different amounts of Tb.

Figure S4 – 3D plot of the excitation and emission properties of CGO: 4% Tb^3+^, CGO: 2% Bi^3+^, 4% Tb^3+^ and CGO: 3% Zn2^+^, 4% Tb^3+^

Figure S5 Thermoluminescence Curves of CGO: 4% Tb^3+^; CGO: 3% Zn^2+^, 4% Tb^3+^; CGO: 2% Bi^3+^, 4% Tb^3+^; samples and fitting curve using the GOK model


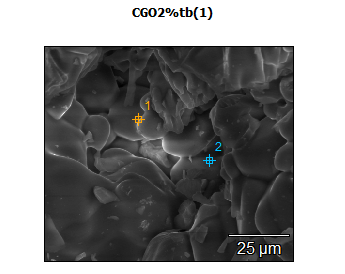


Image Name: CGOBiZn

Image Resolution: 512 by 442

Image Pixel Size: 0.20 µm

Acc. Voltage: 30.0 kV

Magnification: 1300


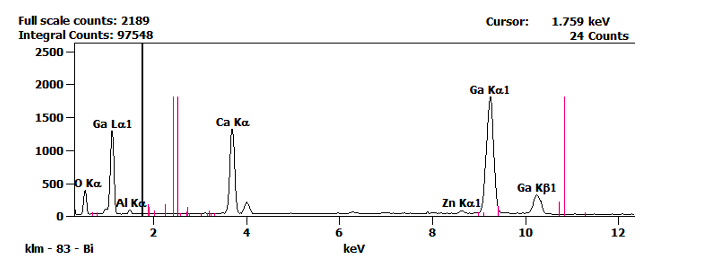


| Element | Weight % | Weight %err | Atom % | Atom % err |
| --- | --- | --- | --- | --- |
| Ca (K) | 21.4 | 0.2 | 18.8 | 0.2 |
| Ga (K) | 48.8 | 0.2 | 24.2 | 0.5 |
| O (K) | 25.9 | 0.5 | 56.1 | 1.3 |
| Tb (L) | 0 | 0.2 | 0 | 0.2 |
| Bi(L) | 2.7 | 0.4 | 0.5 | 0.2 |
| Zn (K) | 1.2 | 0.2 | 0.6 | 0.2 |

Figure S6 – SEM image and elemental analysis of CGO :2%Bi^3+^, 3%Zn^2+^


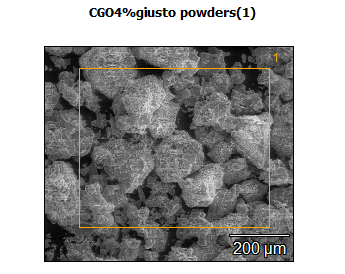


Image Name: CGO4%

Image Resolution: 512 by 442

Image Pixel Size: 1.63 µm

Acc. Voltage: 30.0 kV

Magnification: 160


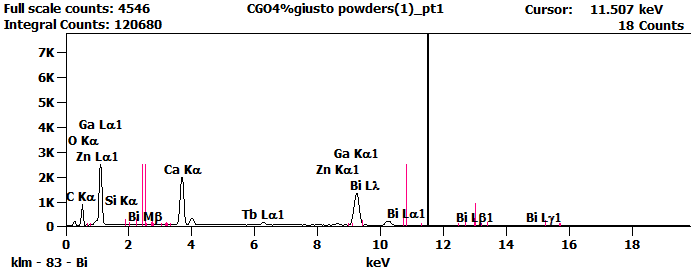


| Element | Weight % | Weight %err | Atom % | Atom % err |
| --- | --- | --- | --- | --- |
| Ca (K) | 16.0 | 0.2 | 17.3 | 0.2 |
| Ga (K) | 40.1 | 0.2 | 25.3 | 0.5 |
| O (K) | 19.2 | 0.5 | 51.9 | 1.3 |
| Tb (L) | 2.4 | 0.2 | 0.7 | 0.2 |
| Bi(L) | 2.1 | 0.4 | 0.4 | 0.2 |
| Zn (K) | 0.9 | 0.2 | 0.6 | 0.2 |

Figure S7 – SEM image and elemental analysis of CGO: 2%Bi ^3+^, 3% Zn^2+^, 4% Tb^3+^
